# Genome-Wide Histone Acetylation Underlies Tumor Intrinsic Immune Signaling Induced by Photothermal Therapy in Ovarian Cancer

**DOI:** 10.21203/rs.3.rs-9144148/v1

**Published:** 2026-04-15

**Authors:** Reddick R. Walker, Melissa Hadley, Jose Colina, Kevin Nestler, Abigail V. Lee, Elizabeth E. Sweeney, Rohan Fernandes, Katherine B. Chiappinelli

**Affiliations:** 1 The George Washington University Cancer Center, Washington, D.C., USA; 2 The George Washington University Microbiology, Immunology and Tropical Medicine Department, Washington, D.C., USA; 3 University of Maryland Fischell Department of Bioengineering, College Park, MD, USA

## Abstract

Ovarian cancers (OCs) remain a lethal gynecological malignancy characterized by an immunosuppressive tumor microenvironment lacking effector lymphocytes. Thus, increasing tumor intrinsic immune signaling in OC remains a therapeutic goal for improving recruitment and activation of lymphocytes to the tumor microenvironment. A nanoparticle-based thermal treatment, Prussian blue nanoparticle photothermal therapy (PBNP-PTT), has shown strong antigen-specific lymphocyte recruitment to the tumor microenvironment through induction of immunogenic cell death in preclinical tumor models. Therefore, this study sought to determine the efficacy of PBNP-PTT in OC cell lines. We first demonstrate that PBNP-PTT induced tumor intrinsic immune signaling in OC cells and led to improved monocyte activation through tumor intrinsic proinflammatory cytokine release and presentation of damage-associated molecular patterns. Accompanying this intrinsic immunostimulatory effect, we report a novel epigenetic hallmark response to PBNP-PTT characterized by genome-wide H3K9 acetylation. Finally, increasing histone acetylation via histone deacetylase inhibition (Panobinostat) improved the tumor intrinsic immune signaling potential of PBNP-PTT. These findings indicate that PBNP-PTT activates the release of immunostimulatory factors in ovarian cancer cells alongside H3K9ac rewiring and suggests the potential of combining PBNP-PTT with HDACi to improve tumor intrinsic immune signaling in ovarian cancer.

## Introduction

A major barrier to the treatment of ovarian cancer (OC) is the lack of effector lymphocytes present in the tumor microenvironment.^1,2^ Clinical outcomes in ovarian cancer clearly show improved survival in the presence of tumor infiltrating lymphocytes, particularly CD8+ T cells.^1,3^ Infiltration of these lymphocytes relies on tumor intrinsic and extrinsic factors including proinflammatory cytokine release and antigen presentation.^4^ OCs are characterized by low neoantigen burden and highly suppressive immune signaling profiles, indicating tumor intrinsic immune signaling is limited in OC.^5^ Unfortunately, OCs do not respond well to immune checkpoint blockade as a single arm agent.^3^

One strategy to improve immune signaling in OC is to promote the release of damage-associated molecular patterns (DAMPs) into the tumor microenvironment to activate immune cells.^6–8^ Immunogenic cell death (ICD) requires 1) the release of DAMPs including HMGB1 and Annexin A1, and 2) presentation of DAMPs on the cell surface including Hsp70 and Calreticulin.^4,9–11^ These signals interact with pattern recognition receptors including TLR2/4 and LRP1 on antigen presenting cells to promote antitumor immunity through uptake of dead cell antigens and active recruitment of effector T lymphocytes.^4^ Thus, the induction of tumor intrinsic immune signaling in OC tumors remains a therapeutic goal to improve patient response to therapy.

We and others have demonstrated the preclinical use of Prussian blue nanoparticle photothermal therapy (PBNP-PTT) of tumor cells induces ICD and improves immune cell recognition and antitumor immunity.^12–17^ This novel thermal therapy uses near infrared, light-sensitive Prussian blue nanoparticles to generate an optimal dose of heat that improves Hsp70 and Calreticulin expression on tumor cells. Using syngeneic neuroblastoma and glioblastoma tumor-bearing mice, these studies indicate *in vivo* PBNP-PTT creates antigen-specific T-cells against the tumor and induces an abscopal effect: destruction of distant metastatic tumor sites from treatment in the primary tumor alone.^13–15^ Therefore, using PBNP-PTT to induce proper activation of tumor intrinsic immune signaling may improve immune responses in OC.

While ICD signaling has been well described,^4,7,18^ little is known about what, if any, epigenetic changes occur during intrinsic tumor immune signaling. The regulation of such immunostimulatory markers, including cytokines and interferon signaling, has become increasingly linked to epigenetic changes.^19^ Indeed, the use of epigenetic therapies including histone deacetylase inhibitors (HDACi) increase transcription of interferons and cytokines to induce anti-tumor immune signaling in various cancers.^20,21^ However, it remains unclear how innate tumor immune signaling itself alters the epigenetic landscape and what, if any, exploitable epigenetic states might exist. To characterize the response of PBNP-PTT in OC cells, we performed PBNP-PTT on four high-grade serous OC cell lines and profiled tumor intrinsic markers of immune signaling alongside multiple epigenetic modifications. We show treatment of OC cells with PBNP-PTT increases markers of ICD alongside induction of a hallmark H3K9ac signature. Finally, we demonstrate that increasing histone acetylation using the FDA approved HDACi, Panobinostat, with PBNP-PTT improves monocyte activation and supports the exploration of this combination treatment in OC.

## Results

### PBNP-PTT induces tumor intrinsic immune signaling in ovarian cancer

**Figure 1:**
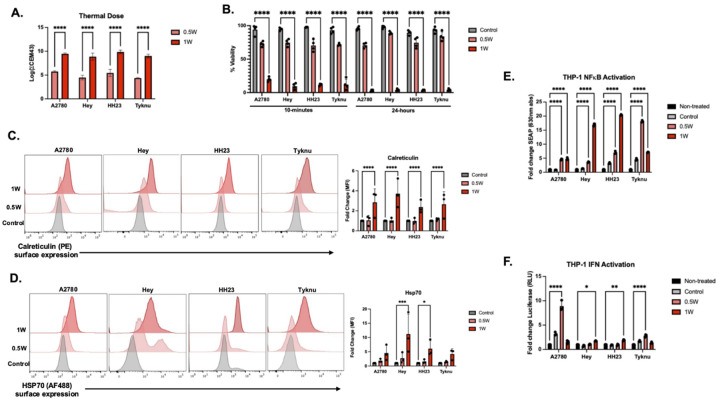
PBNP-PTT induces immunogenic cell death in ovarian cancer A.) Thermal dose at 0.5W and 1W near-infrared laser intensities calculated as the change in Cumulative Equivalent Minutes (CEM) at 43°C for each cell line treated with corresponding laser intensity for ten minutes. B.) Cell viability readings from Trypan blue staining across each time point post-PTT for Prussian blue only (PBO) control (grey) and 1W (red) samples. At least three biological replicates were used. The surface expression of Calreticulin (C.) or Hsp70 (D.) 24-hours post-PTT across three biological replicates for each cell line with corresponding release of cytokines (E and F). Statistics were calculated using a two-way ANOVA with Tukey’s posthoc correction and statistical threshold of p(adj) < 0.05.

Tumor intrinsic immune signaling induced from PBNP-PTT requires an optimal thermal dose that can vary based on the tumor type. To identify this optimal dose, four ovarian cancer (OC) cell lines were treated with PBNP-PTT for ten minutes at 0.5W, 1W, or 1.5W laser intensities (Figure 1A; **S1A, S1B**) or a Prussian blue only (PBO) control. The log-transformed Cumulative Equivalence in Minutes (CEM) at 43°C is used to normalize the total amount of heat generated during the ten minute treatment above the 43°C threshold at which protein unfolding occurs.^22,23^ Immediately following treatment, cell viability significantly dropped below 20% for all cell lines in the 1W condition, decreasing to below 5% after 24 hours (Figure 1B). At 24 hours post treatment, surface expression of both Calreticulin and Hsp70 was significantly increased in the 1W condition across all cell lines, identifying 1W as the optimal thermal dose (Figure 1C, D; **S1C**). To confirm immunostimulatory effects from PBNP-PTT, we co-cultured PBNP-PTT treated tumor cells with dual NFkB/Interferon (IFN) THP-1 reporter monocytes for 24 hours. Co-culture of PBNP-PTT treated tumor cells with THP-1 reporter monocytes resulted in robust monocyte activation compared to tumor cell co-culture alone (Figure 1E, F). NFkB reporter activity was strongly induced across all treated cell lines with comparatively modest IFN pathway activation, suggesting DAMP-mediated signaling from thermal insult engages NFkB-dependent innate immune pathways. Overall, PBNP-PTT treated tumor cells show significantly increased release of proinflammatory signals when treated with the 1W laser intensity. Having established PBNP-PTT induces markers of ICD in OC cells, we next investigated the specific cell death and innate sensing pathways activated by thermal damage.

### PBNP-PTT drives necrotic cell death

**Figure 2:**
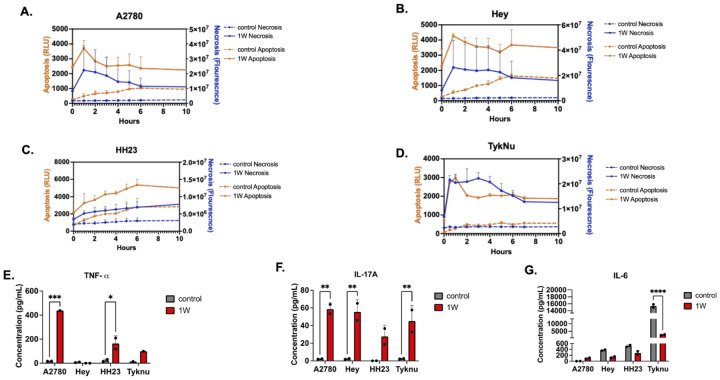
PBNP-PTT drives necrotic cell death Assessment of apoptosis and necrosis was conducted over 10 hours post PTT in the four OC cell lines (A.-D.) using phosphatidyl serine luminescence or cytoplasmic dsDNA fluorescence, respectively. Prussian blue only controls are shown as dotted lines. Corresponding release of cytokines 24 hours post PTT was assessed via tumor cell supernatant Legendplex immunoassay for TNF-a (D.), IL-17A (E.), and IL-6 (F.). At least two biological replicates were used for all studies, and differences in cytokine release were assessed using a 2way ANOVA.

Multiple modes of programmed and nonprogrammed cell death pathways can lead to immunogenic cell death.^4^ Given the amount of heat generated during PBNP-PTT, we tested the extent to which innate sensing pathways triggered by thermal damage to the cell membrane contribute to necrosis and inflammasome signaling. We first profiled apoptosis and necrosis occurrence using exposure of phosphatidyl serine (luminescence) and detection of cytoplasmic nucleic acids (fluorescence), respectively, in the four OC cell lines (Figure 2A–D).^24,25^ Concurrent detection of luciferase-tagged annexin V fusion proteins, which bind exposed phosphatidyl serine, and cell-impermeant fluorescent dsDNA dyes occurred within the first hour post treatment with PBNP-PTT. This suggests tumor cell membrane integrity became acutely impaired, consistent with the necrotic phneotype.^26,27^ Indeed, cytokine profiling revealed elevated release of TNF-a (Figure 2E), a cytokine associated with necroptotic signaling, and IL-33 (**Figure S2A**), a nuclear alarmin passively released upon loss of membrane integrity.^28^ Treatment with PBNP-PTT also increased IL-17A (Figure 2F), IFN-g, and IL-18 release (**Figure S1D, S1E**), cytokines associated with inflammasome signaling. These cytokines are consistent with activation downstream of heat-induced DAMP presentation (Figure 1C, D). Paradoxically, while PBNP-PTT activates NFkB signaling in co-cultured THP-1 cells, IL-6 release from tumor cells was reduced following treatment (Figure 2G), suggesting thermal damage attenuates tumor-intrinsic NFkB transcriptional programs. This indicates immune activation following PBNP-PTT is driven by DAMP-mediated inflammasome signaling rather than tumor cell-derived NFkB dependent cytokines.^29,30^

### PBNP-PTT increases chromatin accessibility via H3K9ac

**Figure 3:**
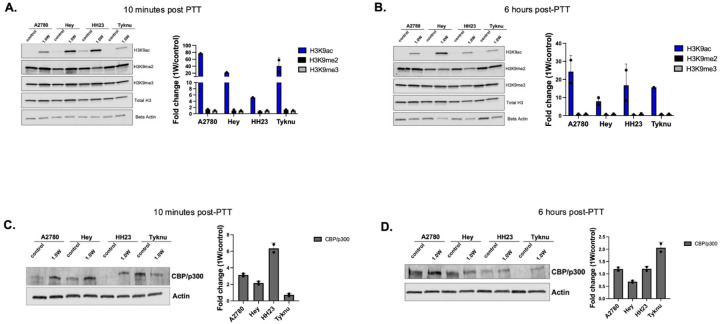
PBNP-PTT induces H3K9ac Changes to histone modifications H3K9ac, H3K9me2, H3K9me3 ten minutes (A.) or six hours (B.) post treatment with PBNP-PTT compared to a Prussian blue only control. Cropped blot images are shown. Inclusion of both Beta Actin and Total H3 loading controls were used and quantification of blots were normalized against Total H3. Fold change was calculated as difference between 1W treatment and control for each cell line. Assessment of the histone acetylation writer, CBP/p300 ten minutes (C.) or six hours (D.) post treatment of PBNP-PTT. Quantification includes Beta actin normalized O.D. signals with fold changes of 1W treatment against control for each cell line.

Activation of genes that regulate thermal insults and the unfolded protein response are regulated by epigenetic modifications made to histone proteins.^31,32^ These modifications can activate (H3K9ac, H3K27ac) or repress (H3K9me2/me3) transcription of target genes, respectively, and represent a major form of transcriptional control.^33,34^ To determine if these epigenetic modifications are induced during PBNP-PTT, we assessed histone modifications in PTT-treated OC cells. In contrast to H3K9me2/me3, which showed little variability between treatment and control samples, H3K9ac was strongly increased by PBNP-PTT immediately following treatment and six hours post treatment (Figure 3A, B). The enzyme responsible for deposition of H3K9ac, p300/CBP, similarly increases upon treatment with PBNP-PTT (Figure 3C, D). To map where these acetylation gains occur genome-wide, we next profiled H3K9ac genome regions using CUT&RUN-sequencing.

### Induced H3K9ac peaks mark chromatin remodeling genes

**Figure 4:**
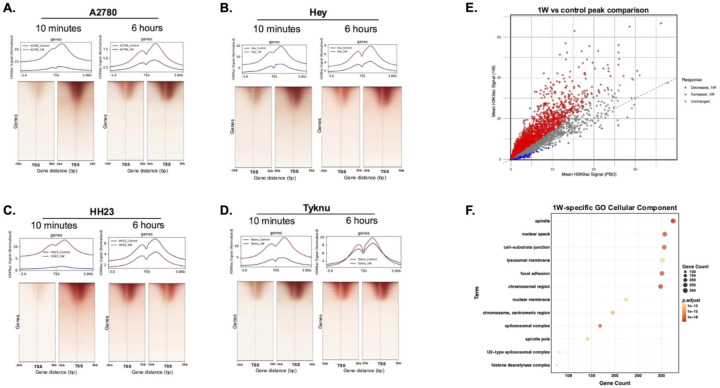
Genome-wide H3K9ac marks 1W-specific gene promoters Two biological replicates of H3K9ac CUT&RUN for each condition were combined using the bedtools merge function. (A. -D.) Corresponding bigwigs were generated using deeptools computeMatrix with TSS reference-point across all Gencode annotated genes. E.) 6-hour H3K9ac CUT&RUN peaks were combined across the four cell lines into 1W and Prussian blue only control groups using bedtools intersect. F.) Top 500 1W-specific peaks were run through gene ontology for cellular component processes.

To understand the extent to which surviving tumor cells coordinate a regulated acetylation response after PBNP-PTT, we performed H3K9ac CUT&RUN-sequencing which relies on intact nuclei to probe for protein-DNA interactions. In alignment with western blot results, we observe a strong increase of H3K9ac in our 1W treatment group, predominantly localized around the transcription start site (TSS) (Figure 4A–D). Increased H3K9ac signal at the transcription start site (TSS) is crucial for increasing accessibility of transcriptional machinery necessary for proper RNA polymerase II extension during transcription.^35^

We next identified the 6-hour 1W specific H3K9ac peaks by combining treatment-specific peaks across our four OC cell lines and comparing these against control conditions (Figure 4E). Taking the top 500 genes with highest gain in H3K9ac, we ran a gene ontology analysis to identify processes associated with the gained acetylated genes. The top enriched pathways included chromosomal region and histone deacetylase complex (HDAC) cellular components (Figure 4F). Despite acetylation of key genes involved in apoptosis, necrosis and NFkB signaling (**Figure S3A, B**), we did not see significant transcriptional activation of these genes (**Figure S3C**). Additionally, secreted cytokines induced by PBNP-PTT, including IL-17A, did not show clear H3K9ac gain (**Figure S3D**), consistent with the DAMP-mediated, NFkB independent cytokine release observed in Figure 2.

### H3K9ac mediates transcription of heat shock response

**Figure 5:**
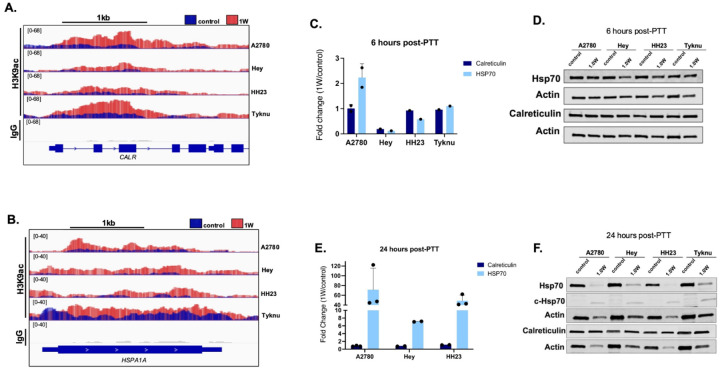
H3K9ac mediates transcription of Hsp70 Gain of H3K9ac at either the *CALR* (A.) or *HSPA1A* (B.) genes from CUT&RUN-seq data. Two biological replicate bigwigs for each cell line and treatment were combined using bedtools merge. Tracks were visualized using IGV. qRT-PCR of *CALR* and *HSPA1A* gene expression (C.) and corresponding protein expression (D.) six hours post-PTT. 24-hour expression changes of qRT-PCR (E.) and protein (F.) qRT-PCR fold change is calculated as 1W over PBO control and normalized against beta actin with at least two biological replicates. Standard deviation error bars are shown.

We next asked whether the gain in H3K9ac was driving the response to the thermal injury generated by our treatment. We compared 1W and control PBO treated H3K9ac levels at *CALR* and *HSPA1A* gene loci. While Calreticulin and Hsp70 DAMPs both showed strong acetylation gain in 1W-treated conditions six hours post-PTT (Figure 5A, B), this active chromatin mark did not correlate with increased RNA expression at the same time point (Figure 5C). Consistent with the RNA, we did not see changes to the protein expression of these genes at six hours (Figure 5D). However, 24 hours post treatment analysis of Hsp70 RNA expression greatly increased post-PTT (Figure 5E), indicating H3K9ac priming at six hours activates heat shock response gene transcription at a later timepoint. Interestingly, protein expression of full-length Hsp70 at 24 hours was markedly decreased with a ~30kDa cleaved Hsp70 band dominating the treatment group (Figure 5F).

### Combination HDACi treatment improves PBNP-PTT induced ICD

Previous nanoparticle-mediated *in vivo* experiments have shown reductions in tumor progression when combined with histone deacetylase (HDAC) inhibitors.^36^ Given the strong gain in H3K9ac seen in our treatment, we next questioned if pre-treatment with an HDACi would improve the response to PBNP-PTT. We reasoned that blocking this response to elevated H3K9ac using the FDA-approved HDAC inhibitor, Panobinostat^37^, might lead to improved ICD through prolonged acetylation of the genome.

**Figure 6:**
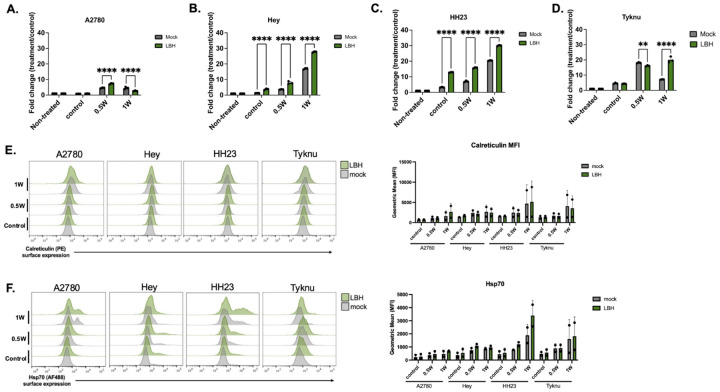
Combination HDACi treatment improves PBNP-PTT induced ICD Cells were treated with 100nM of Panobinostat for six hours followed by PBNP-PTT treatment. Corresponding samples were co-cultured with THP-1 reporter monocytes for 24 hours immediately following PTT treatment and collection of SEAP absorbance in the four OC cell lines (A-D.) Surface expression of Calreticulin (E.) or Hsp70 (F.) was assessed 24 hours post PTT treatment. At least two biological replicates were used for all experiments. Statistics were calculated using a two-way ANOVA with Tukey’s posthoc correction and statistical threshold of p(adj) < 0.05.

Pre-treating the tumor cells for six hours with Panobinostat before treatment with PBNP-PTT significantly improved monocyte activation at 1W in all cell lines but A2780 (Figure 6A–D). This indicates that increasing chromatin accessibility before treatment with PBNP-PTT sensitizes the cells to lower dose thermal therapy. Despite their lack of IFN-b (SEAP) induction, pre-treatment of the A2780 cell line showed a stronger ISG-54 (luciferase) response in THP-1 cells at the 0.5W laser intensity (**Figure S6A**).

To determine the extent to which pretreatment of tumor cells with Panobinostat induces surface DAMP expression, we repeated the drug treatment and PBNP-PTT administration and collected samples 24 hours post treatment to assess surface expression of Calreticulin and Hsp70. Interestingly, while pretreatment with Panobinostat increased THP1 activation, the pretreatment did not significantly change the surface expression of Calreticulin or Hsp70 (Figure 6E, F). Overall, activation from coculture experiments indicate the cells are more immunogenic in the combination treated group, but this is independent from surface expression of the ICD markers Calreticulin and Hsp70.

## Discussion

PBNP-PTT is a novel treatment modality that increases lymphocyte activation and antitumor immune responses in preclinical neuroblastoma and glioblastoma cancer models.^12,15^ This response is mediated by the induction of immunogenic cell death (ICD) that releases factors to recruit and activate effector immune cells.^4,7^ We aimed to clarify the efficacy of PBNP-PTT in ovarian tumors with an emphasis on the epigenetic responses to treatment. Our findings reveal two mechanistically distinct responses to PBNP-PTT: a rapid, epigenetics-independent immunostimulatory program driven by membrane disruption, DAMP release, and inflammasome cytokine profiles, and an epigenetic remodeling program mediated by genome-wide H3K9 acetylation that primes heat shock gene transcription.

Ovarian cancers remain a therapeutic challenge due to poor proinflammatory response in the tumor microenvironment, underscoring the need for strategies that improve tumor intrinsic immune signaling.^3^ PBNP-PTT treatment of OC tumor cell lines increases presentation of Calreticulin and Hsp70 DAMPs and release of proinflammatory cytokines including TNF-a, IL-18, and IFN-g. Functionally, these signals robustly activate monocytes, with NFkB reporter induction substantially stronger than IFN pathway activation. These results suggest MyD88-dependent innate sensing pathways by Hsp70 and inflammasome-derived cytokines signaling through TLR2/4 and IL-18R, respectively.^38,39^ Importantly, this immune cell NFkB activation occurs despite suppression of tumor-intrinsic NFkB transcriptional programs evidenced by reduced IL-6. This is consistent with reports of heat shock factors negatively regulating NFkB signaling and suggests that PBNP-PTT may be advantageous to maintaining proinflammatory DAMP/inflammasome signaling while reducing production of pro-tumorigenic cytokines like IL-6.^30,40^

The genome-wide H3K9ac gain from PBNP-PTT represents a novel thermal response that has not been previously reported. Interestingly, we see increases of CBP/p300 protein expression as early as ten minutes post treatment with PBNP-PTT. This timescale would be too fast to be driven by transcriptional changes to CBP/p300 and likely is due to posttranslational modifications made to the protein, including phosphorylation, which stabilizes p300 expression.^41,42^ While H3K9ac gain occurs broadly across the genome, including at loci involved in apoptosis, necrosis, and NFkB signaling, transcriptional activation at six hours is selective rather than global. The most prominent transcriptional consequence of H3K9ac priming is a delayed upregulation of Hsp70, a critical mediator of both thermal protection and ICD. Decreased full-length Hsp70 protein and accumulation of a ~30kDa cleaved fragment suggests active processing and release of Hsp70 as a DAMP signal, consistent with the sustained surface expression observed in Figure 1. By increasing histone acetylation with HDACi (Panobinostat), we show higher activation of monocytes in lower thermal doses indicating the combination treatment improves OC responses to PBNP-PTT. Together, these findings establish a framework in which thermal therapy and epigenetic modulation cooperate through distinct but complementary mechanisms to overcome immunologically barren microenvironment characteristics of OCs.

## Methods

### Cell Lines

Human ovarian carcinoma cell lines (A2780, Hey, HH23, and Tyknu) were cultured in RPMI1640 (Corning, 10–104-CV) with 10% FBS (X&Y Cell Culture, FBS-500-HI) and 1% penicillin/streptomycin solution (Gibco, 15070063). The A2780, Hey, and Tyknu cell lines were kind gifts from Dr. Stephen Baylin, Johns Hopkins University School of Medicine. The HH23 cell line is a CRISPR cas9 R175H-modified cell line derived from the Hey parental as previously described.^43^ Cells were cultured in T175 dishes (Greiner, 82050–872) and stored in 37°C, 5% CO_2_ incubators. Cell lines were periodically tested for *Mycoplasma* using the Lonza MycoAlert kit (VWR, 75870–454).

For the HDAC inhibitor treatment, cells were grown until 75% confluent in T-75 flasks and treated with 100 nmol/L Panobinostat (Selleckchem, S1030) or DMSO for 6 hours before treatment with PBNP-PTT.

### Prussian Blue Photothermal Therapy

#### Synthesis:

Prussian blue nanoparticles (PBNPs) were synthesized as previously described.^44^ Briefly, an aqueous solution of Fe(III)Cl_3_ hexahydrate (Sigma, 236489) was combined with aqueous K4(II)Fe(CN)_6_ trihydrate (Sigma, P3289) and stirred for 15 minutes to form nanoparticles. The nanoparticles were isolated using centrifugation (20,000 × g, 10 minutes) and resuspended in sterile Milli-Q water (Millipore Corporation). The resuspended particles were sonicated using the Q500 probe sonicator (QSonica LLC) and the corresponding particle size and surface charge was measured using a Zetasizer Nano ZS (Malvern Instruments).

#### Treatment:

Cell lines were collected from flasks using CellStripper (Corning, MT25056CI) and resuspended in 1.5 mL Eppendorf tube at a concentration of 10e^6^ cells/mL in a 0.5 mL suspension of culture media. PBNP-PTT was performed using an 808 nm NIR laser (Laserglow) with PBNPs at a final concentration of 0.15 mg/mL for 10 minutes. Following this treatment, samples were either immediately pelleted and frozen at −80°C for protein isolation or replated in T-75 flasks (Greiner, 658170) until specified time point. Replated cells were collected using CellStripper, washed once in sterile 1X PBS (Gibco, 10-010-049), and either collected for flow cytometry, pelleted for protein/RNA collection, or viably frozen (90% FBS, 10% DMSO) in cryotubes for CUT&RUN.

### Western Blot Analysis

Cells were lysed in RIPA buffer (Pierce, 89900) with 1X protease and phosphatase inhibitor (Pierce, A32961). Lysates were exposed to sonication at 4°C in a water bath Bioruptor (Diagenode) for 8 cycles of 30 seconds on and 30 seconds off on the high amplitude setting. Following this, samples were centrifuged at 4°C, 10,000 × g for 10 minutes to pellet cellular debris. Protein concentrations were measured using the Pierce BCA Protein Assay Kit (Thermo Fisher Scientific, 23225) and 20 mg of isolated protein was mixed with NuPage LDS 4x loading gel (NP0007) and NuPAGE 10x reducing agent (NP0009) before heating at 100 C for 10 minutes. SDS-PAGE was run using 4%−20% gradient gels (Bio-Rad, 4561093) and transferred on LF PVDF (Bio-Rad, 170–4274) membranes. Next, the membranes were blocked using LI-COR Biosciences Intercept Blocking Buffer (VWR, 927–70001) for 2 hours and room temperature, followed by an overnight 4°C incubation with the primary antibodies and Total H3 housekeeping. Blots that used -actin as a housekeeping received a 30-minute room temperature incubation the day following overnight primary staining. All antibody dilutions and catalogue numbers are listed in Supplementary Table S1. The secondary antibodies used were AzureSpectra700 (AC2128) and AzureSpectra800 (AC2135). Membranes were imaged using the Azure Biosystems Imaging System c600. Processing of the images was performed using the LI-COR Biosciences Image Studio software.

### qRT-PCR

Cells were resuspended in 1 mL of TRIzol reagent (ThermoFisher, 15596018) and frozen at −80°C overnight. Following this, RNA was washed three times using chloroform (ThermoFisher, AC167735000) and precipitated at 20°C overnight in isopropyl alcohol (ThermoFisher, 327272500). RNA was then pelleted by 4°C centrifugation (15,000 rpm, 30 minutes) and washed three times in ethanol (Fisher Scientific, BP2818500). After air drying the RNA pellets at room temperature for 8 minutes, RNA was resuspended in nuclease-free water (Qiagen, 129117) and concentrations were measured using a nanodrop. 1 mg of RNA was DNase-treated (Thermo, EN0525) for 30 minutes at 37°C. EDTA was added to quench the reaction at a final concentration of 4.6 mM and the DNase enzyme was denatured by heating for 10 minutes at 65°C. RNA was then reverse transcribed into cDNA using the Applied Biosystems High-Capacity cDNA Reverse Transcription Kit (4368814) and incubated at 25°C for 10 minutes, 27°C for 120 minutes, 85°C for 5 minutes, followed by a 4°C hold. qRT-PCR was performed with Taqman reagents (Fisher Scientific, 4369510) on the QuantStudio3 Quantitate reverse transcriptase PCR system. All qRT-PCR primer sequences and catalogue numbers are listed in Supplementary Table S1. At least two biological replicates plated in three technical triplicates were included for all qRT-PCR experiments.

### Flow cytometry surface staining

Cells were stained with Live/dead Fixable Aqua (Invitrogen, L34965) at a 1:500 dilution in 1X PBS for 30 minutes at room temperature. Surface expression of Calreticulin and Hsp70 were assessed using Enzo Calreticulin-PE conjugate (ADI-SPA-601PE-D, 1:1000) and Biolegend Hsp70-AF488 (648003, 1:5000) staining at 4°C for 30 minutes. Data was acquired using a BD FACS Celesta and analyzed using FlowJo v10.8.1 with manual compensation using single color controls and UltraComp eBeads (ThermoFisher, 01-2222-41). Statistics were calculated using a two-way ANOVA with Tukey’s posthoc correction and statistical threshold of p(adj) < 0.05.

### Legendplex immunoassay

5×10^6^ cells/mL cells were treated with PBNP-PTT for 10 minutes and re-plated into 10 cm^2^ dishes for 24 hours. Cell medium was collected and centrifuged at 500 × g for 5 minutes. 1mL supernatant was moved to clean 1.5 mL microcentrifuge tubes and stored at −80°C. Supernatant was thawed and 25 mL was plated into V-bottom 96-well plates (Corning, 2610). Quantification of secreted cytokines was performed with the Human Inflammation Panel I Legendplex Immunoassay (Biolegend, 740809) according to manufacturer’s protocol. At least two replicates were used and statistical testing for cytokine analysis between control and 1W group was conducted using a 2way ANOVA with a posthoc Sidak’s multiple comparisons test.

### CUT&RUN

CUT&RUN was carried out using the Cell Signaling Technologies CUT&RUN kit (86652) as previously described.^45^ Briefly, 500,000 cells were resuspended in 1X wash buffer and bound to Concanavalin A-coated magnetic beads for 5 minutes at room temperature. Samples were split into PCR tubes and resuspended in antibody binding buffer. Samples were then incubated with the primary antibody at a 1:50 dilution overnight at 4°C. The next day, samples were washed twice with a 1X digitonin wash buffer and then incubated with Protein A-MNase at 4°C for 1 hour. Samples were washed again with a 1X digitonin wash buffer and then incubated for 30 minutes at 4°C with CaCl_2_ to activate the Protein A-MNase and digest DNA fragments. An equal volume of 1X stop buffer containing (X) *S. Cerevisiae* spike-in DNA was added to each sample and then subsequently heated at 37°C for 10 minutes to release DNA fragments. Resulting supernatants were collected and extracted using the CUTANA DNA Purification Kit (Epicypher, 14–0050). The purified DNA was subsequently prepared for sequencing libraries using the NEBNext Ultra II library preparation kit (NEB, E7645S).

### CUT&RUN-sequencing data processing

#### Sequencing:

Prepared CUT&RUN DNA library size distribution and molar concentration was determined using an Agilent 4200 TapeStation. 5 million reads of 50×50bp paired-end sequences was performed on the Illumina NextSeq 2000 platform by the George Washington University Genomics Core. Raw fastq files were analyzed on the George Washington University High Computing Server, as described previously by the Henikoff lab.^46^

#### Trimming:

Adaptor sequences were trimmed using cutadapt v1.18 with parameters “-q 20

-minimum-length 1

-a AGATCGGAAGAGCACACGTCTGAACTCCAGTCAC

-A AGATCGGAAGAGCGTCGTGTAGGGAAAGAGTGT”

#### Alignment:

To align trimmed reads, we used Bowtie2 v2.5.3 to the hg38 genome reference sequence from UCSC with parameters “-t -end-to-end -no-mixed -no-discordant”. *S. Cerevisiae* reads were mapped to the sacCer3 genome reference sequence from UCSC with the same bowtie2 parameters as above.

#### Analysis and Visualization:

Spike-in calibration was performed by calculating a scale factor that divides 1,000,000 by the number of spike-in mapped reads. Spike-in normalized alignment bam files were converted to bigwig files using deeptools bamCoverage. Genomic bigwig tracks were displayed using the Integrated Genome Viewer (IGV). Heatmaps were generated using deepTools v3.5.6 computeMatrix and plotHeatmap functions. Gene Ontology (GO) enrichment analysis was performed using the clusterProfiler package v4.8.2 with parameters “pAdjustMethod = “BH” qvalueCutoff = 0.05” and visualized with the dotplot function in ggplot2 v3.5.2. For each CUT&RUN experiment, two biological replicates were included.

### Co-incubation of PBNP-PTT treated tumors with THP1-Dual reporter monocytes

Post-treated tumor cells were co-cultured with THP-1 dual reporter monocytes (InvivoGen, catalogue: thpd-nfis) at a 1:1 ratio in normal cell culture medium for 24 hours as previously described.^47^ Activation of NF-kB-SEAP and IRF-Lucia luciferase in reporter monocytes was measured by secreted alkaline phosphatase (SEAP) in a 96-well plate and luciferase production in a white bottom 96-well plate, respectively. SEAP expression was measured by incubating the QUANTI-Blue solution with each co-culture sample for 2 hours followed by optical density reading at 630 nm using a Spectramax i3X microplate reader. Luciferase induction was measured by placing the 1X QUANTI-Luc 4 reagent into each co-culture sample and immediately reading an end-point luminescence using a microplate reader. At least two biological replicates plated in three technical triplicates were used for each experiment.

### In vitro apoptosis assay

To assess apoptosis and necrosis of PBNP-PTT treated cells in vitro, PBNP-PTT was administered to 5×10^6^ cells for 10 min. 2×10^4^ cells were then seeded in 96-well white-walled flat-bottom plates. Accumulation of apoptotic cells was quantified with RealTime-Glo Annexin V Apoptosis and Necrosis Assay (Promega Cat #JA1011) according to the manufacturer’s protocol. Statistics were calculated using a two-way ANOVA with Tukey’s posthoc correction and statistical threshold of p(adj) < 0.05.

## Data Availability

Sequencing data generated in this study have been deposited in the NCBI Gene Expression Omnibus (GEO) database under accession number GSE326102. (https://www.ncbi.nlm.nih.gov/geo/query/acc.cgi?acc=GSE326102) Code used for downstream processing of sequencing files can be found on the github page (https://github.com/redwalk/epiPTT-cutandrun-project).

## References

[1] Sato E. Intraepithelial CD8+ tumor-infiltrating lymphocytes and a high CD8+/regulatory T cell ratio are associated with favorable prognosis in ovarian cancer. Proc. Natl. Acad. Sci. U. S. A. 102, 18538–18543 (2005). 16344461 10.1073/pnas.0509182102 PMC1311741

[2] Zhang L. Intratumoral T Cells, Recurrence, and Survival in Epithelial Ovarian Cancer. N. Engl. J. Med. 348, 203–213 (2003). 12529460 10.1056/NEJMoa020177

[3] Gupta R., Kumar R., Penn C. A. & Wajapeyee N. Immune evasion in ovarian cancer: implications for immunotherapy and emerging treatments. Trends Immunol. 46, 166–181 (2025). 39855990 10.1016/j.it.2024.12.006 PMC11835538

[4] Galluzzi L., Buqué A., Kepp O., Zitvogel L. & Kroemer G. Immunogenic cell death in cancer and infectious disease. Nat. Rev. Immunol. 17, 97–111 (2017). 27748397 10.1038/nri.2016.107

[5] Matulonis U. A. Ovarian cancer. Nat. Rev. Dis. Primer 2, 16061 (2016). 10.1038/nrdp.2016.61 PMC7290868 27558151

[6] Nagata S. & Tanaka M. Programmed cell death and the immune system. Nat. Rev. Immunol. 17, 333–340 (2017). 28163302 10.1038/nri.2016.153

[7] Catanzaro E., Beltrán-Visiedo M., Galluzzi L. & Krysko D. V. Immunogenicity of cell death and cancer immunotherapy with immune checkpoint inhibitors. Cell. Mol. Immunol. 22, 24–39 (2025). 39653769 10.1038/s41423-024-01245-8 PMC11685666

[8] Hänggi K. & Ruffell B. Cell death, therapeutics, and the immune response in cancer. Trends Cancer 9, 381–396 (2023). 36841748 10.1016/j.trecan.2023.02.001 PMC10121860

[9] Galluzzi L. Essential versus accessory aspects of cell death: recommendations of the NCCD 2015. Cell Death Differ. 22, 58–73 (2015). 25236395 10.1038/cdd.2014.137 PMC4262782

[10] Peng F. Regulated cell death (RCD) in cancer: key pathways and targeted therapies. Signal Transduct. Target. Ther. 7, 286 (2022). 35963853 10.1038/s41392-022-01110-y PMC9376115

[11] Park W. Diversity and complexity of cell death: a historical review. Exp. Mol. Med. 55, 1573–1594 (2023). 37612413 10.1038/s12276-023-01078-x PMC10474147

[12] Sweeney E. E., Cano-Mejia J. & Fernandes R. Photothermal Therapy Generates a Thermal Window of Immunogenic Cell Death in Neuroblastoma. Small 14, 1800678 (2018). 10.1002/smll.201800678 29665282

[13] Sweeney E. E. Photothermal Prussian blue nanoparticles generate potent multi-targeted tumor-specific T cells as an adoptive cell therapy. Bioeng. Transl. Med. 9, e10639 (2024). 38818122 10.1002/btm2.10639 PMC11135148

[14] Sweeney E. E. Engineered tumor-specific T cells using immunostimulatory photothermal nanoparticles. Cytotherapy 25, 718–727 (2023). 37278683 10.1016/j.jcyt.2023.03.014 PMC10264146

[15] Sweeney E. E. ENGINEERING AUTOLOGOUS GLIOBLASTOMA-SPECIFIC T CELLS USING NANOPARTICLE-BASED PHOTOTHERMAL THERAPY. Cytotherapy 26, S13 (2024).

[16] Balakrishnan P. B., Sweeney E. E., Ramanujam A. S. & Fernandes R. Photothermal therapies to improve immune checkpoint blockade for cancer. Int. J. Hyperthermia 37, 34–49 (2020). 33426992 10.1080/02656736.2020.1797190 PMC7808273

[17] Tang K. Recent advances in Prussian blue-based photothermal therapy in cancer treatment. https://doi.org/10.1039/D3BM00509G (2023) doi:10.1039/D3BM00509G. 37067845

[18] Dou L., Fang Y., Yang H., Ai G. & Shen N. Immunogenic cell death: A new strategy to enhancing cancer immunotherapy. Hum. Vaccines Immunother. 20, 2437918 (2024). 10.1080/21645515.2024.2437918 PMC11639453 39655738

[19] Cruickshank B. Dying to Be Noticed: Epigenetic Regulation of Immunogenic Cell Death for Cancer Immunotherapy. Front. Immunol. 9, (2018). 10.3389/fimmu.2018.00654 PMC5891575 29666625

[20] Cao J. & Yan Q. Cancer Epigenetics, Tumor Immunity, and Immunotherapy. Trends Cancer 6, 580–592 (2020). 32610068 10.1016/j.trecan.2020.02.003 PMC7330177

[21] Modulation of Antitumor Immunity with Histone Deacetylase Inhibitors: Immunotherapy: Vol 9, No 16. https://www.tandfonline.com/doi/abs/10.2217/imt-2017-0134. 10.2217/imt-2017-0134 PMC6077764 29185390

[22] Sapareto S. A. & Dewey W. C. Thermal dose determination in cancer therapy. Int. J. Radiat. Oncol. 10, 787–800 (1984). 10.1016/0360-3016(84)90379-1 6547421

[23] Full article: Quantification of thermal dose in moderate clinical hyperthermia with radiotherapy: a relook using temperature-time area under the curve (AUC). https://www.tandfonline.com/doi/full/10.1080/02656736.2021.1875060. 10.1080/02656736.2021.1875060 33627018

[24] Nakamura T. Cytosolic Double-Stranded DNA as a Damage-Associated Molecular Pattern Induces the Inflammatory Response in Rat Pancreatic Stellate Cells: A Plausible Mechanism for Tissue Injury-Associated Pancreatitis. Int. J. Inflamm. 2012, 504128 (2012). 10.1155/2012/504128 PMC3328960 22550608

[25] Fadok V. A., Bratton D. L., Frasch S. C., Warner M. L. & Henson P. M. The role of phosphatidylserine in recognition of apoptotic cells by phagocytes. Cell Death Differ. 5, 551–562 (1998). 10200509 10.1038/sj.cdd.4400404

[26] Berghe T. V. Necroptosis, necrosis and secondary necrosis converge on similar cellular disintegration features. Cell Death Differ. 17, 922–930 (2010). 20010783 10.1038/cdd.2009.184

[27] Guo X. NFκB promotes oxidative stress-induced necrosis and ischemia/reperfusion injury by inhibiting Nrf2-ARE pathway. Free Radic. Biol. Med. 159, 125–135 (2020). 32745764 10.1016/j.freeradbiomed.2020.07.031 PMC7530060

[28] Cayrol C. & Girard J.-P. Interleukin-33 (IL-33): A nuclear cytokine from the IL-1 family. Immunol. Rev. 281, 154–168 (2018). 29247993 10.1111/imr.12619

[29] Liu T., Zhang L., Joo D. & Sun S.-C. NF-κB signaling in inflammation. Signal Transduct. Target. Ther. 2, 17023 (2017). 29158945 10.1038/sigtrans.2017.23 PMC5661633

[30] Paszek A. Heat shock response regulates stimulus-specificity and sensitivity of the pro-inflammatory NF-κB signalling. Cell Commun. Signal. 18, 77 (2020). 32448393 10.1186/s12964-020-00583-0 PMC7245923

[31] Muñoz-Carvajal F. & Sanhueza M. The Mitochondrial Unfolded Protein Response: A Hinge Between Healthy and Pathological Aging. Front. Aging Neurosci. 12, 581849 (2020). 33061907 10.3389/fnagi.2020.581849 PMC7518384

[32] Periyasamy P. & Shinohara T. Age-Related Cataracts: Role of unfolded protein response, Ca2+ mobilization, epigenetic DNA modifications, and loss of Nrf2/Keap1 dependent cytoprotection. Prog. Retin. Eye Res. 60, 1–19 (2017). 28864287 10.1016/j.preteyeres.2017.08.003 PMC5600869

[33] Esteller M. The Epigenetic Hallmarks of Cancer. Cancer Discov. 14, 1783–1809 (2024). 39363741 10.1158/2159-8290.CD-24-0296

[34] Walker R. R., Rentia Z. & Chiappinelli K. B. Epigenetically programmed resistance to chemo- and immuno-therapies. Adv. Cancer Res. 158, 41–71 (2023). 36990538 10.1016/bs.acr.2022.12.001 PMC10184181

[35] Gates L. A. Acetylation on histone H3 lysine 9 mediates a switch from transcription initiation to elongation. J. Biol. Chem. 292, 14456–14472 (2017). 28717009 10.1074/jbc.M117.802074 PMC5582839

[36] Ledezma D. K. Indocyanine Green-Nexturastat A-PLGA Nanoparticles Combine Photothermal and Epigenetic Therapy for Melanoma. Nanomaterials 10, 161 (2020). 31963449 10.3390/nano10010161 PMC7022377

[37] Dickinson M. Preliminary evidence of disease response to the pan deacetylase inhibitor panobinostat (LBH589) in refractory Hodgkin Lymphoma. Br. J. Haematol. 147, 97–101 (2009). 19663825 10.1111/j.1365-2141.2009.07837.x

[38] Goodyear A., Troyer R., Bielefeldt-Ohmann H. & Dow S. MyD88-Dependent Recruitment of Monocytes and Dendritic Cells Required for Protection from Pulmonary Burkholderia mallei Infection. Infect. Immun. 80, 110–120 (2012). 22025508 10.1128/IAI.05819-11 PMC3255660

[39] Owen A. M. MyD88-dependent signaling drives toll-like receptor-induced trained immunity in macrophages. Front. Immunol. 13, (2022). 10.3389/fimmu.2022.1044662 PMC9692127 36439136

[40] Fisher D. T., Appenheimer M. M. & Evans S. S. The Two Faces of IL-6 in the Tumor Microenvironment. Semin. Immunol. 26, 38–47 (2014). 24602448 10.1016/j.smim.2014.01.008 PMC3970580

[41] Huang W.-C. & Chen C.-C. Akt Phosphorylation of p300 at Ser-1834 Is Essential for Its Histone Acetyltransferase and Transcriptional Activity. Mol. Cell. Biol. 25, 6592–6602 (2005). 16024795 10.1128/MCB.25.15.6592-6602.2005 PMC1190347

[42] Yazıcı E. & McIntyre J. The complex network of p300/CBP regulation: Interactions, posttranslational modifications, and therapeutic implications. J. Biol. Chem. 301, 110715 (2025). 40962055 10.1016/j.jbc.2025.110715 PMC12547938

[43] McDonald J. I. Epigenetic Therapies in Ovarian Cancer Alter Repetitive Element Expression in a TP53-Dependent Manner. Cancer Res. 81, 5176–5189 (2021). 34433584 10.1158/0008-5472.CAN-20-4243 PMC8530980

[44] Dumont M. F. Biofunctionalized Gadolinium-Containing Prussian Blue Nanoparticles as Multimodal Molecular Imaging Agents. Bioconjug. Chem. 25, 129–137 (2014). 24328306 10.1021/bc4004266

[45] Skene P. J. & Henikoff S. An efficient targeted nuclease strategy for high-resolution mapping of DNA binding sites. eLife 6, e21856 (2017). 28079019 10.7554/eLife.21856 PMC5310842

[46] Wu W., Ahmad K. & Henikoff S. Chromatin-bound U2AF2 splicing factor ensures exon inclusion. Mol. Cell 85, 1982–1998.e4 (2025). 40315850 10.1016/j.molcel.2025.04.013 PMC13075997

[47] Immunostimulatory activity of inactivated environmental Bacillus isolates and their endospores | Scientific Reports. https://www.nature.com/articles/s41598-025-12833-7. 10.1038/s41598-025-12833-7 PMC12368082 40835982

